# Do the UK government's new Quality and Outcomes Framework (QOF) scores adequately measure primary care performance? A cross-sectional survey of routine healthcare data

**DOI:** 10.1186/1472-6963-7-166

**Published:** 2007-10-17

**Authors:** Amy Downing, Gavin Rudge, Yaping Cheng, Yu-Kang Tu, Justin Keen, Mark S Gilthorpe

**Affiliations:** 1 Cancer Epidemiology Group, Centre for Epidemiology & Biostatistics, University of Leeds, 30-32 Hyde Terrace, Leeds, LS2 9LN, UK; 2 Health Services Research, Department of Public Health & Epidemiology, University of Birmingham, Edgbaston, Birmingham, B15 2TT, UK; 3 Biostatistics Unit, Centre for Epidemiology & Biostatistics, University of Leeds, 30-32 Hyde Terrace, Leeds, LS2 9LN, UK; 4 Centre for Health & Social Care, University of Leeds, Leeds, LS2 9PL, UK

## Abstract

**Background:**

General practitioners' remuneration is now linked directly to the scores attained in the Quality and Outcomes Framework (QOF). The success of this approach depends in part on designing a robust and clinically meaningful set of indicators. The aim of this study was to assess the extent to which measures of health observed in practice populations are correlated with their QOF scores, after accounting for the established associations between health outcomes and socio-demographics.

**Methods:**

QOF data for the period April 2004 to March 2005 were obtained for all general practices in two English Primary Care Trusts. These data were linked to data for emergency hospital admissions (for asthma, cancer, chronic obstructive pulmonary disease, coronary hear disease, diabetes, stroke and all other conditions) and all cause mortality for the period September 2004 to August 2005. Multilevel logistic regression models explored the association between health outcomes (hospital admission and death) and practice QOF scores (clinical, additional services and organisational domains), age, sex and socio-economic deprivation.

**Results:**

Higher clinical domain scores were generally associated with lower admission rates and this was significant for cancer and other conditions in PCT 2. Higher scores in the additional services domain were associated with higher admission rates, significantly so for asthma, CHD, stroke and other conditions in PCT 1 and cancer in PCT 2. Little association was observed between the organisational domain scores and admissions. The relationship between the QOF variables and mortality was less clear. Being female was associated with fewer admissions for cancer and CHD and lower mortality rates. Increasing age was mainly associated with an increased number of events. Increasing deprivation was associated with higher admission rates for all conditions and with higher mortality rates.

**Conclusion:**

The associations between QOF scores and emergency admissions and mortality were small and inconsistent, whilst the impact of socio-economic deprivation on the outcomes was much stronger. These results have implications for the use of target-based remuneration of general practitioners and emphasise the need to tackle inequalities and improve the health of disadvantaged groups and the population as a whole.

## Background

Over the course of the last twenty years, doctors have become used to reviewing the quality of their clinical work. We are in an era of performance management, where the quality of doctors' work is judged by external bodies, and doctors are being offered financial incentives to achieve quality and other targets set by commissioners of services. The new general medical services contract, covering the work of general practitioners (GPs) in the UK National Health Service (NHS), includes clinical performance targets in the GPs' employment contracts. GPs' remuneration is now linked directly to the attainment of targets contained in a set of indicators called the Quality and Outcomes Framework (QOF) [1].

The QOF indicators are divided into four domains: clinical, organisational, patient experience, and additional services [2,3] (See Table 1 for a summary of the domains and their indicators). The clinical domain indicators apply to clinical or clinically driven processes and are arranged into disease specific groups. Some measure the extent of particular interventions, such as the proportion of diagnosed coronary heart disease (CHD) patients taking aspirin, an alternative antiplatelet therapy, or an anti-coagulant. Often, the indicators cover the administrative tasks which support effective clinical management, such as maintenance of disease registers. The organisational domain is designed to reflect the quality of basic administrative processes, the patient experience domain largely measures processes around the administration of the annual patient survey, and the additional services domain covers a range of specific additional services offered by some practices.

**Table 1:** Summary of the indicators included in each QOF domain

**Additional services domain**
Cervical screening
Child health surveillance
Contraception
Maternity services

**Clinical domain**

Asthma
Atrial fibrillation
Cancer
Chronic kidney disease
Chronic obstructive pulmonary disease
Dementia
Depression
Diabetes mellitus
Epilepsy
Heart failure
Hypertension
Hypothyroidism
Learning difficulties
Mental health
Obesity
Palliative care
Secondary prevention in coronary heart disease
Smoking indicators
Stroke & transient ischaemic attacks

**Organisation domain**

Education & training
Information for patients
Medicines management
Practice management
Records & information about patients

**Patient experience domain**

Length of consultation
Patients surveys

The success or failure of this target-based approach depends in significant part on two issues, namely whether it is possible to design a robust and clinically meaningful set of indicators, and whether financial incentives change doctors' behaviour and thereby improve patient care. The aim of this study is to address the first of these issues, by assessing the extent to which the UK government's new QOF scores are correlated with measures of health observed in practice populations, after accounting for well-established associations between health outcomes and demographic and socio-economic factors. The outcome measures considered were those that were readily obtainable from routinely collected data, namely mortality and hospital admission.

## Methods

### Data extraction and linkage

Two neighbouring Primary Care Trusts (PCTs) formed the target study population, with approximately 360,000 (PCT 1) and 157,000 (PCT 2) individuals resident in and registered with a GP in the same PCT. At the time of collection, after the first year of the QOF, data from only two PCTs were available. The PCTs are situated in the West Midlands region of the UK and cover mainly urban areas. QOF data for the 68 GP practices in PCT 1 and 26 practices in PCT 2 for the period April 2004 to March 2005 were obtained from the PCTs. Data from the clinical, organisational and additional services domains were used. The patient experience domain lacked sufficient variation to be informative (there were only five distinct scores across the two PCTs, with most GPs achieving identical scores of 100). The additional services domain did not vary much also, with most values clustered close to the maximum of 36. Nevertheless, sufficient variation existed to be informative and this domain was retained in the analyses (See Table 2 for summary characteristics of the QOF domains).

**Table 2:** Median, range and quartile range of the QOF domain and Index of Multiple Deprivation income domain scores by PCT

	**PCT 1 (n = 359,863)**			**PCT 2 (n = 156,757)**		
**Model covariate**	**Median**	**Range**	**Quartile range**	**Median**	**Range**	**Quartile range**
Additional services	35.8	21.9 – 36.0	0.50	36.0	14.8 – 36.0	0.36
Clinical	531.5	291.7 – 549.9	13.03	545.9	469.0 – 550.0	11.70
Organisational	180.0	58.5 – 184.0	8.00	183.0	116.5 – 184.0	3.50
Patient experience	100.0	0.0 – 100.0	0.00	100.0	70.0 – 100.0	0.00
Income domain	0.18	0.02 – 0.70	0.10	0.10	0.01 – 0.60	0.09

Using data supplied to the PCTs from secondary care providers, information on emergency admissions occurring between September 2004 and August 2005 were derived by practice. Associations between elective admissions and process measures are complex. Elective admission is more vulnerable to service factors, such as admission procedures within individual hospitals and waiting list management. Modelling these factors would involve creating a further level, that of hospital, which was not within the scope of this study. The admissions were grouped by main primary diagnosis into: asthma, cancer, CHD, chronic obstructive pulmonary disease (COPD), diabetes, stroke, and all other conditions. Information on mortality in the study population within the same period was also obtained from the PCT data. In addition, patient age (in five-year bands) and sex were extracted. A geographical measure of socio-economic deprivation was obtained by matching the income domain scores of the Index of Multiple Deprivation 2004 [4] to the census-based super output area (SOA) of residence. The income domain includes measures such as the number of adults and children in Income Support or Job Seekers Allowance households. This domain was preferred to the total score, as the total includes measures of access to healthcare. These data were then linked with the GP practice QOF scores according to the lists held by the PCT (i.e. all patients listed under the same GP practice would be linked to the QOF scores for that practice).

### Statistical analysis

The data structure is one of complex clustering, both within health care systems (where patients are nested within GPs within PCTs) and within geographical areas (where patients are nested within SOAs). In addition, both hierarchies are crossed, i.e. not strictly nested within one another. To address these complexities, we used cross-classified multilevel logistic regression [5], using the software *MLwiN *[6], which allows covariates (explanatory variables) to be incorporated in the model 'operating' at the correct level of the system hierarchy. For instance, deprivation (area-level covariate) is correctly specified to impact across the nested level of SOAs, whilst the QOF scores (GP-level covariate) are correctly specified to impact across the nested level of GPs. Although a measure of socio-economic deprivation is assigned to each patient, within a multilevel modelling framework these scores impact (correctly) at the area-level, avoiding the problem of the ecological fallacy [7]. The total outcome variation is partitioned into that between patients but *within *GPs and that between patients but *within *areas, which indicates to what extent the unexplained variation within the model pertains to differences across either GPs or areas.

We calculated odds ratios (ORs) with 95% credible intervals (CIs) for all cause mortality and emergency admissions for the disease groupings listed above. All models included six covariates specified at three levels: 5-year age group, sex (both at the patient-level), three QOF domain scores (GP-level), and income domain score (area-level), whilst deriving the proportion of unexplained variance at each cluster level. QOF achievement was incorporated using the total points within each domain, rather than disease-specific scores, as many of the indicators could impact upon multiple disease areas. To facilitate comparison across the QOF domains, each was scaled (a form of standardisation) such that their increased or decreased odds correspond to a quartile change in the score (e.g. a transition from the lower quartile to the median, or from the median to the upper quartile). The income domain score was similarly scaled. Table 2 summarises the quartile ranges to which these covariates were scaled.

## Results

In PCT 1, 14.3% of the population were aged over 65 years compared to 17.4% in PCT 2. Males made up 50.3% and 49.2% of the populations of PCT 1 and 2 respectively. As a whole, PCT 1 was more deprived than PCT 2 (median income domain score of 0.18 versus 0.10). The median clinical domain scores were 531.5 in PCT 1 and 545.9 in PCT 2, the median organisational domain scores were 180 in PCT 1 and 176 in PCT 2, and the median additional services domain scores were 35.8 in PCT 1 and 36.0 in PCT 2.

### Hospital admissions

Tables 3 and 4 show the results of the models for emergency admissions for the two PCTs. Higher additional services domain scores were significantly associated with increased admissions for asthma, CHD, stroke and other conditions in PCT 1 and cancer in PCT 2. Increasing clinical domain scores were generally associated with reduced admissions, although this was only significant for cancer and other conditions in PCT 2 (OR 0.86; 95% CI 0.79–0.93 and OR 0.94; 95% CI 0.92–0.97 per quartile increase in score respectively). There was no clear association between the organisational domain and hospital admissions.

**Table 3:** Associations between patient, area and GP level covariates and hospital admissions and mortality in PCT 1

	**Emergency admission**			
	
	**Asthma**	**Cancer**	**COPD**	**CHD**
	**(N = 386)**	**(N = 566)**	**(N = 542)**	**(N = 819)**
**Patient-level; OR (95% CI)**				
Female	1.42 (1.17–1.75)	0.68 (0.57–0.80)	0.90 (0.76–1.07)	0.56 (0.48–0.64)
5-year age group	1.01 (0.99–1.03)	1.38 (1.34–1.41)	1.45 (1.42–1.49)	1.41 (1.38–1.44)
**Area-level; OR (95% CI)**				
Income domain*	1.23 (1.14–1.32)	1.07 (1.00–1.14)	1.38 (1.29–1.48)	1.15 (1.08–1.22)
**GP-level; OR (95% CI)**				
Additional Services*	1.04 (1.01–1.08)	1.00 (0.97–1.03)	1.02 (0.98–1.05)	1.03 (1.01–1.07)
Clinical*	1.05 (0.96–1.14)	0.99 (0.92–1.05)	0.99 (0.92–1.07)	0.97 (0.91–1.03)
Organisational*	0.95 (0.89–1.03)	1.02 (0.95–1.09)	0.98 (0.91–1.05)	0.98 (0.92–1.03)
**Variance (95% CI)**				
Area-level	0.00 (0.00–0.03)	0.00 (0.00–0.02)	0.11 (0.00–0.19)	0.04 (0.01–0.11)
GP-level	0.01 (0.00–0.05)	0.01 (0.00–0.07)	0.02 (0.00–0.08)	0.01 (0.00–0.05)

	**Emergency admission**			**Mortality**
	
	**Diabetes**	**Stroke**	**Other**	**All causes**
	**(N = 164)**	**(N = 360)**	**(N = 18,784)**	**(N = 3,201)**

**Patient-level; OR (95% CI)**				
Female	0.81 (0.60–1.12)	0.91 (0.74–1.12)	1.10 (1.06–1.13)	0.69 (0.64–0.75)
5-year age group	1.13 (1.09–1.17)	1.55 (1.49–1.61)	1.14 (1.13–1.14)	1.68 (1.65–1.70)
**Area-level; OR (95% CI)**				
Income domain*	1.21 (1.08–1.36)	1.10 (1.01–1.19)	1.14 (1.12–1.16)	1.10 (1.06–1.14)
**GP-level; OR (95% CI)**				
Additional Services*	1.03 (0.98–1.10)	1.05 (1.01–1.11)	1.03 (1.01–1.04)	1.01 (0.99–1.03)
Clinical*	0.94 (0.83–1.05)	1.00 (0.92–1.10)	0.99 (0.96–1.03)	0.97 (0.92–1.01)
Organisational*	1.03 (0.92–1.16)	0.96 (0.89–1.04)	0.98 (0.95–1.02)	1.02 (0.98–1.07)
**Variance (95% CI)**				
Area-level	0.01 (0.00–0.21)	0.00 (0.00–0.02)	0.01 (0.00–0.01)	0.05 (0.03–0.07)
GP-level	0.02 (0.00–0.16)	0.01 (0.00–0.04)	0.04 (0.03–0.06)	0.03 (0.01–0.06)

N = the number of admissions/deaths (out of 359,863); *Results per quartile increase in score

**Table 4:** Associations between patient, area and GP level covariates and hospital admissions and mortality in PCT 2

	**Emergency admission**			
	
	**Asthma**	**Cancer**	**COPD**	**CHD**
	**(N = 35)**	**(N = 250)**	**(N = 197)**	**(N = 318)**
**Patient-level; OR (95% CI)**				
Female	1.96 (0.99–4.17)	0.74 (0.57–0.94)	0.92 (0.70–1.23)	0.54 (0.43–0.68)
5-year age group	0.92 (0.85–1.00)	1.38 (1.33–1.44)	1.43 (1.37–1.50)	1.38 (1.34–1.43)
**Area-level; OR (95% CI)**				
Income domain*	1.27 (1.00–1.58)	1.14 (1.03–1.25)	1.42 (1.28–1.57)	1.16 (1.07–1.26)
**GP-level; OR (95% CI)**				
Additional Services*	0.99 (0.96–1.04)	1.03 (1.01–1.05)	1.00 (0.98–1.03)	1.00 (0.98–1.01)
Clinical*	0.87 (0.71–1.06)	0.86 (0.79–0.93)	0.94 (0.86–1.02)	1.00 (0.93–1.09)
Organisational*	0.96 (0.90–1.04)	1.00 (0.97–1.04)	1.05 (1.01–1.09)	1.01 (0.98–1.04)
**Variance (95% CI)**				
Area-level	0.04 (0.00–0.58)	0.00 (0.00–0.12)	0.01 (0.00–0.06)	0.01 (0.00–0.04)
GP-level	0.02 (0.00–0.87)	0.01 (0.00–0.17)	0.01 (0.00–0.13)	0.01 (0.00–0.06)

	**Emergency admission**			**Mortality**
	
	**Diabetes**	**Stroke**	**Other**	**All causes**
	**(N = 58)**	**(N = 160)**	**(N = 6,847)**	**(N = 1,423)**

**Patient-level; OR (95% CI)**				
Female	0.72 (0.42–1.22)	0.85 (0.62–1.16)	1.10 (1.05–1.16)	0.71 (0.64–0.80)
5-year age group	1.15 (1.09–1.23)	1.62 (1.52–1.72)	1.12 (1.11–1.12)	1.69 (1.66–1.73)
**Area-level; OR (95% CI)**				
Income domain*	1.15 (0.95–1.38)	1.17 (1.03–1.31)	1.13 (1.10–1.16)	1.11 (1.06–1.17)
**GP-level; OR (95% CI)**				
Additional Services*	0.99 (0.96–1.02)	1.02 (1.00–1.05)	1.01 (1.00–1.01)	1.01 (0.99–1.02)
Clinical*	1.11 (0.92–1.37)	0.95 (0.86–1.07)	0.94 (0.92–0.97)	0.98 (0.92–1.05)
Organisational*	0.98 (0.92–1.06)	0.99 (0.95–1.04)	1.00 (1.00–1.01)	0.99 (0.97–1.01)
**Variance (95% CI)**				
Area-level	0.02 (0.00–0.11)	0.01 (0.00–0.08)	0.01 (0.00–0.02)	0.02 (0.00–0.06)
GP-level	0.02 (0.00–0.44)	0.02 (0.00–0.19)	0.01 (0.00–0.02)	0.04 (0.01–0.13)

N = the number of admissions/deaths (out of 156,758); *Results per quartile increase in score

Within both PCTs, females had significantly fewer admissions than males for cancer and CHD, but females were more likely to be admitted for asthma and other conditions. Age was positively associated with all admissions in both PCTs, except those for asthma. Higher deprivation scores were associated with an increased likelihood of admission for all conditions. Those most impacted upon by deprivation were COPD (PCT 1 OR 1.38; 95% CI 1.29–1.48 and PCT 2 OR 1.42; 95% CI 1.28–1.57 per quartile increase in income domain score), and asthma (PCT 1 OR 1.23; 95% CI 1.14–1.32 and PCT 2 OR 1.27; 95% CI 1.00–1.58 per quartile increase).

The area-level and GP-level variance terms refer to the amount of unexplained variation at each of these levels. A figure of zero, or close to zero, means that there is little or no residual variation, i.e. all the variation at that level has been explained. Generally, the variances were small, both at the area-level and GP-level, meaning that there is little unexplained variation at both levels.

### All cause mortality

None of the QOF variables were significantly associated with mortality in either PCT. As expected, increasing age was significantly associated with increased mortality, whilst being female was significantly associated with decreased mortality (in both PCTs). Increasing deprivation was associated with significantly increased mortality (PCT 1 OR 1.10, 95% CI 1.06–1.14 and PCT 2 OR 1.11, 95% CI 1.06–1.17 per quartile increase in income domain score). As with emergency admissions, there was little unexplained variation in mortality at the area- and GP-levels.

## Discussion

This study provides an early insight into the relationship between QOF scores and patient outcomes measured in terms of emergency hospital admissions and mortality. Whilst there were several associations between QOF and the outcome measures, these were not consistent. Generally, the results suggest a greater association between the outcomes and deprivation than with any of the QOF scores. However, when significant associations between deprivation and the outcome measures were observed, there was still some unexplained variation at the SOA level, suggestive of unmodelled factors operating within the patient (area-level) environment. These include levels of crime, education and unemployment and the availability of leisure facilities. A study in Canada found that health correlated more frequently with non-medical determinants (i.e. health behaviours and living and working conditions) than healthcare performance indicators [8].

Looking in more detail at the QOF measures, higher clinical domain scores were generally associated with lower odds of unplanned admission. Activities such as the maintenance of disease registers, high levels of prescribing and opportunistic screening may be driving this in high scoring practices. However, the associations were not consistent and were significant only for cancer and other conditions (in PCT 2). The fact that performance indicators are associated with small effects on outcomes has been reported previously [9]. Unsurprisingly, there was no clear association with outcomes for the domain that has less obvious relevance to admissions and mortality, the organisational domain. There was an association between higher scores in the additional services domain and increased odds of unplanned admission for a number of disease groups. The additional services covered in the indicator set are largely aimed at younger patient groups (e.g. child health surveillance, emergency contraception, maternity services), whereas, the outcomes captured in this study (admission and death) are concentrated amongst older people. It is possible that higher levels of additional services lead to a resource/time competition between the care of young people and care of the elderly and to higher admissions/mortality in the elderly. However, this is speculation and we are unable to explain the increase in admissions for specific disease groups. Mortality was associated with the socio-demographic variables in line with expectation. However, mortality showed no significant relationship with any of the QOF domains. Whilst both admission and mortality will be associated with long-term factors, of the two, admission rates may be more likely to be modified by short term interventions than mortality.

We should not be surprised by some lack of consistency in the associations found in this study. The complexity of the relationship between processes and outcomes in healthcare has been known for some time, as illustrated by Donabedian in 1966 [10]. Perhaps, as an acknowledgement of the complexity with which variables operate in community settings, the author of this early work specifically excluded community care delivery from the scope of the paper. More recent work on how different kinds of indicator operate has provided a critique of quality assessment based upon process indicators [11]. This work highlights the extent to which process variables are confounded by the effects of factors such as deprivation and case mix. This supports the use of a hierarchical approach for investigating the relationships between process and outcome whilst adjusting for confounders operating at different levels.

Few studies using QOF data have been published, although those that have suggest that the current indicators do not adequately measure quality of care. One study looked at the correlation between six performance indicators, one of which was the QOF, for the 303 PCTs in England [12]. They found little correlation, except in the specific area of screening, and suggested that current indicators do not have sufficient construct validity to measure the underlying concept of quality. Another more focused study investigated whether higher QOF scores were associated with better stroke care, assessed using the Royal College of Physicians (RCP) guidelines [13]. Higher stroke quality scores did not reflect better adherence to RCP guidance and it was concluded that further research is needed to assess the generalisability of this finding and how the QOF might be better aligned with delivering best practice.

The distribution of the scores in all domains was clustered towards the upper limit of their maximum, which leads us to question to what extent the QOF scores are a sensitive measure of quality of care. It may be that disaggregating the scores and focusing upon some of the higher level measures within the domains is preferable. Another option would be to use percentage achievement. Indicators for which the targets are more difficult to achieve tend to have lower maximum thresholds and so more variation may be found using percentage achievement than by the points awarded.

A recent article by Guthrie and colleagues suggests that the value of the QOF could be improved by incorporating treatment data in to the quality indicators, as the current targets fail to identify clear opportunities for improving healthcare, thereby leading to therapeutic inertia [14]. A practice may be able to achieve maximum QOF payment, for example by fulfilling the criteria of a certain proportion of patients having a blood pressure below a certain level, despite missed opportunities for tighter control such as the prescribing of new antihypertensive drugs or increasing the treatment dose where appropriate.

### Strengths and limitations

The key question underpinning this work is whether or not the processes that the QOF scores measure will result in measurable changes in the populations' health. The results suggest that this does not appear to be the case. The main limitation of this study is the short lag time between the implementation of the QOF and measurement of the health outcomes, which may not be sufficient to assess the full impact of the QOF. However, the risks of hospital admission or death are a function of factors that have been operating over a period of time. Whilst most of these factors operate at the individual level (such as those related to lifestyle), some will have impacted at the practice level (such as the propensity to refer for secondary interventions, for example coronary artery by-pass operation). It will be some time before the longer-term impact of the QOF can be measured.

One feature of the QOF design is that it allows GPs to exclude certain patients from the performance calculations for specific indicators (exception reporting), for example those with terminal illness, but there is a possibility that GPs may inappropriately exclude patients where they have failed to meet the targets in order to artificially improve their performance. This may weaken any associations between the QOF scores (based on a selected population) and the health of the responsible population (without selection). Doran and colleagues looked at this and found a significant positive relationship between rates of exception reporting and reported achievement for certain clinical indicators, although, the effect was small with a 1% increase in the proportion of excluded patients leading to a 0.31% increase in reported achievement [15]. McLean and colleagues found that the exception reporting system contributed to a continuation of the inverse care law [16]. For quality indicators based on care delivered to all patients there was no evidence of worse care in deprived areas. However, for indicators allowing the exclusion of patients 17 of the 33 measures showed markedly worse care for more deprived populations. It is unclear how exception reporting affects the results of our study.

Due to the novelty of these indicators, data from only two PCTs were available for this initial exploration of how the target and indicator approach contributes to improving and maintaining the quality of patient care. Ideally, one would wish to examine many more PCTs to establish if consistent patterns of association exist. Also, it must be stated that the PCTs included in this study were more deprived than the "average" PCT in England. When compared against the Healthcare Commission's list of PCTs ranked by Index of Multiple Deprivation score, PCT 1 would fall in decile 8 and PCT 2 in decile 6 (where decile 10 represents the most deprived PCTs in the country). A question for further study would be whether the patterns of association between the QOF scores and the outcomes differ by level of deprivation.

## Conclusion

The results of this study show that the associations between practice QOF scores and health outcomes were small and inconsistent, whilst the relationship between socio-economic deprivation and health was much stronger. This has implications for the use of target-based remuneration of GPs and other doctors, because it suggests that the assumptions about process-outcome links underpinning such schemes may be flawed. Despite some limitations, these results highlight the point that the clinical value of the QOF is dependent on the selection of the indicators, and at present, this process does not seem to have generated as sensitive a set of indicators as intended. The importance of socio-economic deprivation in explaining theobserved variation in outcomes highlights the key role of public health and health promotion, and emphasises the need to tackle inequalities and improve the health of disadvantaged groups and the population as a whole.

## Competing interests

The author(s) declare that they have no competing interests.

## Authors' contributions

MSG and GR designed the study. AD undertook the data linkage and managed the data. YC, YKT, and AD analysed the data. MSG, AD and GR wrote the first draft of the paper. All authors contributed to the final draft.

## Pre-publication history

The pre-publication history for this paper can be accessed here:



## References

[B1] Statutory Instrument 291 (2004). The National Health Service (General Medical Services Contracts) Regulations 2004: Sch3.

[B2] Department of Health (2003). Investing in General Practice; The New General Medical Services Contract.

[B3] Department of Health (2004). Updated version of the QOF guidance and evidence base.

[B4] Office of the Deputy Prime Minister (2005). The English Indices of Deprivation 2004: Summary (revised).

[B5] Leyland AH, Boddy FA (1998). League tables and acute myocardial infarction. Lancet.

[B6] Rasbash J, Browne WJ, Healy M, Cameron B, Charlton C (2005). MLwiN version 202.

[B7] Robinson WS (1950). Ecological Correlations and the Behavior of Individuals. Am Sociol Rev.

[B8] Arah OA, Westert GP (2005). Correlates of health and healthcare performance: applying the Canadian health indicators framework at the provincial-territorial level. BMC Health Serv Res.

[B9] Werner RM, Bradlow ET (2006). Relationship between Medicare's Hospital Compare performance measures and mortality rates. J Am Med Assoc.

[B10] Donabedian A (1966). Evaluating the quality of healthcare. Millbank memorial fund quarterly.

[B11] Mant J (2001). Process versus outcome indicators in the assessment of quality of health care. Int J Qual Health Care.

[B12] Brown CA, Lilford RJ (2006). Cross sectional study of performance indicators for English Primary Care Trusts: testing construct validity and identifying explanatory variables. BMC Health Serv Res.

[B13] Williams PH, de Lusignan S (2006). Does a higher 'quality points' score mean better care in stroke? An audit of general practice medical records. Inform Prim Care.

[B14] Guthrie B, Inkster M, Fahey T (2007). Tackling therapeutic inertia: role of treatment data in quality indicators. Brit Med J.

[B15] Doran T, Fullwood C, Gravelle H, Reeves D, Kontopantelis E, Hiroeh U, Roland M (2006). Pay-for-performance programs in family practices in the United Kingdom. New Engl J Med.

[B16] McLean G, Sutton M, Guthrie B (2006). Deprivation and quality of primary care services: evidence for persistence of the inverse care law from the UK Quality and Outcomes Framework. J Epidemiol Comm Health.

